# Birds as reservoirs: unraveling the global spread of Gamma- and Deltacoronaviruses

**DOI:** 10.1128/mbio.02324-24

**Published:** 2024-09-04

**Authors:** Jinyue Guo, Jieheng He, Zhaoping Liang, Shujian Huang, Feng Wen

**Affiliations:** 1 College of Life Science and Engineering, Foshan University, Foshan, Guangdong, China; 2 College of Veterinary Medicine, South China Agricultural University, Guangzhou, Guangdong, China; The Ohio State University, Columbus, Ohio, USA

**Keywords:** coronavirus, *Gammacoronaviruses*, *Deltacoronaviruses*, waterfowl, wild birds, cross-species transmission

## Abstract

Avian migration is a global phenomenon that transcends geographical boundaries. These migratory birds serve as unwitting carriers of diverse Gammacoronaviruses (γ-CoVs) and Deltacoronaviruses (δ-CoVs). While recombination events have been documented among γ-CoVs in avian species and β-CoVs in mammals, evidence for recombination between CoVs of distinct genera remains limited. This minireview examines the prevalence of CoVs in both domestic waterfowl (ducks and geese) and wild bird populations inhabiting various regions. We investigate the dissemination patterns of γ-CoVs and δ-CoVs among these populations, highlighting their shared characteristics. Furthermore, the review explores the intricate web of cross-species transmission of δ-CoVs from wild birds to mammals, with a particular focus on pigs. Understanding the distinct features of CoVs harbored by waterfowl and wild birds and their potential for cross-species transmission is crucial for preparedness and response to future CoV epidemics.

## INTRODUCTION

The COVID-19 pandemic, caused by the SARS-CoV-2 virus, has had a profound impact on global health, resulting in over 6.7 million deaths as of 21 January 2023 (1). Studies highlight the significant role of birds as hosts for various viruses, which can be transmitted within avian populations before potentially spilling over to humans through intermediate hosts (2). This underscores the urgency of monitoring coronavirus (CoV) prevalence in animal populations and investigating cross-species transmission mechanisms to prevent future public health emergencies.

Coronaviruses were first isolated from chicks with laryngotracheitis and cultivated in chicken embryos in the 1930s (3, 4). The *Coronaviridae* family was formally recognized by the International Committee on Taxonomy of Viruses (ICTV) in 1975. The ninth ICTV report (2011) further classified the subfamily *Orthocoronavirinae* into four distinct genera: *Alphacoronavirus* (α-CoV), *Betacoronavirus* (β-CoV), *Deltacoronavirus* (δ-CoV), and *Gammacoronavirus* (γ-CoV) (5). This classification was based on rooted phylogeny and calculated pairwise evolutionary distances of seven conserved domains within the replicase polyprotein (pp1ab). Phylogenetic outliers are considered a new genus if they share less than 46% amino acid sequence identity in these conserved replicase domains with established family members. Members of the same species must exhibit greater than 90% amino acid identity within these domains (5). Notably, CoV genomes are among the largest identified RNA viruses, spanning approximately 26–32 kb (6). The structural features of γ-CoVs and δ-CoVs are similar to other CoVs. Both genera possess single-stranded RNA genomes enveloped in a membrane derived from the host cell during viral assembly. δ-CoVs have a relatively smaller genome size of 25.4 kb, while γ-CoVs have a larger genome of 27.6 kb (7).

The spike (S) protein, characterized by its distinctive corona-like morphology visible in electron microscopy images, is the primary surface protein of CoVs. It plays a crucial role in mediating viral attachment to host cells, facilitating the fusion of the viral membrane with the cell membrane, and enabling viral entry into host cells (8). Composed of two distinct subunits, S1 and S2, the S protein binds to the virus receptor through its S1 subunit, while the S2 subunit mediates membrane fusion (8). Notably, the S protein is the most variable surface protein among CoVs, contributing to antigenic variation and potential immune evasion. Neutralizing antibodies and cell-mediated immune responses against the S protein are essential for protective immunity and the development of effective vaccines against CoVs (9).

The membrane (M) protein and envelope (E) protein, integral components of the viral envelope, play critical roles in viral particle assembly and release (10, 11). The nucleocapsid (N) protein, on the other hand, is crucial for various aspects of viral replication, transcription, and assembly through its interactions with both viral and host cell proteins (12–14).

Domestic waterfowl and wild birds (herein, “wild birds” refer to non-domesticated species inhabiting natural environments) have been implicated in the interspecies transmission of zoonotic viruses, such as H5N1 avian influenza virus and West Nile virus (15). These birds can act as reservoirs for viruses, facilitating their spread to other animals, including humans. This minireview focuses on elucidating the epidemiological characteristics of γ-CoVs and δ-CoVs in domestic waterfowl and wild birds and exploring potential mechanisms involved in their transmission across different species.

## DETECTION AND EPIDEMIOLOGY OF CoVs IN DOMESTIC WATERFOWL AND WILD BIRDS

In terrestrial poultry, γ-CoVs are primarily associated with infectious bronchitis (IB) and turkey viral enteritis (16, 17). In contrast, CoVs found in domestic waterfowl predominantly consist of species-specific γ-CoVs, such as duck CoVs and goose CoVs. While initially classified as γ-CoVs due to limited genomic data, these waterfowl CoVs wer e recognized as distinct variants from those found in terrestrial poultry. However, the updated CoV species classification by the 2022 ICTV (18) now formally recognizes two subgenera (*Brangacovirus* and *Igacovirus*) and two species (*Gammacoronavirus brantae* and *Gammacoronavirus anatis*) associated with waterfowl species within the γ-CoV genus.

Globally, γ-CoVs are widely distributed among domestic waterfowl and wild birds across all continents, except Antarctica. While extensive research has focused on identifying CoVs in avian populations worldwide (19, 20), the clinical manifestations of γ-CoV infections in domestic waterfowl and other wild bird species remain poorly understood. This knowledge gap is likely attributed to limitations in sample collection methods (primarily focused on fecal samples from domestic waterfowl and wild birds) and detection techniques.

In 2005, Jonassen et al. (21) detected CoVs by reverse transcription-polymerase chain reaction (RT-PCR) in fecal and cloacal samples collected from mallard ducks (*Anas platyrhynchos*), Greylag geese (*Anser anser*), and pigeons (*Columba livia*). In 2013, a novel CoV, designated DK/CH/ZJ2012 (KM454473), was discovered in Chinese ducks using metagenomic methods (22). Comparative analysis of three conserved replicase amino acid domains of this virus with 13 infectious bronchitis virus (IBV) sequences revealed mean identities of 87.30% for 1b1, 86.37% for 1b2, and 86.66% for 1b3. In contrast, the amino acid sequence identities between an earlier duck CoV, DK/CH/HN/ZZ2004 (JF705860), and the same 13 IBVs in 1b1, 1b2, and 1b3 were 95.99%, 95.35%, and 97.31%, respectively. Based on ICTV demarcation criteria, DK/CH/HN/ZZ2004 was classified as the same species as IBV, whereas DK/CH/ZJ2012 was likely a novel species.

In 2014, Zhuang et al. (23) conducted large-scale surveillance for CoVs in ducks and identified a highly prevalent CoV that they designated duck-dominant coronavirus (DdCoV). Subsequently, this group expanded surveillance to multiple avian species and found three CoVs associated with pigeons, ducks, and chickens (24). These viruses were categorized into distinct phylogenetic lineages, suggesting a tendency for host-specific circulation. Despite its name, duck CoVs are not restricted to ducks, as related sequences have been detected in a variety of waterfowl species (25). Asymptomatic carriage of γ-CoVs in domestic waterfowl and wild birds poses a risk for long-distance transmission and dissemination of these viruses. Zhuang et al. (23, 24) found a higher prevalence of CoVs in live poultry markets compared to farms and natural habitats, likely due to the concentration of avian species in these markets. However, this finding may be specific to certain regions or countries with significant centralized live poultry markets, such as China.

In 2017, a significant goose mortality outbreak occurred in the Cambridge Bay area of Canada, and a CoV named Canada goose-Branta canadensis-coronavirus-Cambridge Bay 2017 (BcanCoVCB17) was identified from cloacal swabs (26). Studies indicate that goose CoVs are likely transmitted through the fecal–oral route, supported by their detection in a significant proportion of cloacal swab samples, with the intestine identified as the primary site for CoV replication in geese (21). However, direct experimental infection data are limited, and the relative importance of this route compared to other routes (e.g., respiratory transmission) remains unclear. Further research is needed to investigate CoV shedding patterns in different body fluids and determine the most significant transmission routes in goose populations. Interestingly, the virus has been detected in fresh goose feces collected during both spring and autumn, suggesting that goose CoVs may persist in domestic waterfowl for extended periods and remain infectious throughout most of the year. However, no clinical signs were observed in wild ducks infected with goose CoVs.

## DIVERSITY OF δ-CoVs IN BIRDS

The δ-CoVs genus encompasses three subgenus: *Andecovirus, Buldecovirus*, and *Herdecovirus*, and seven species. Of these, six species are exclusively found in birds, while CoV HKU15 is present in both birds and mammals (27). These viruses were initially discovered in southern China in 2006, where they were detected in wild Asian leopards and Chinese ferret badgers (28). Subsequent studies have revealed the wide prevalence of δ-CoVs in various species of wild birds (29–32). Between 2009 and 2012, Woo et al. identified seven different species of δ-CoVs in birds and pigs (33). In 2014, a novel porcine deltacoronavirus (PDCoV) strain, HKU15 OH1987, was identified in pigs exhibiting gastrointestinal symptoms in the United States (34). The exact timeline and mechanisms underlying the emergence and spread of PDCoV within pig herds remain unclear. However, research suggests that birds are the primary source of PDCoV genes, playing a significant role in the evolution and dissemination of δ-CoVs (33, 35).

Compared to domestic waterfowl γ-CoV infections, δ-CoVs have a higher prevalence in pigs and terrestrial birds. While δ-CoVs have been reported in wild waterfowl populations worldwide, isolation of the virus from birds and their excretions has been limited. The majority of complete genome sequences available on NCBI pertain to terrestrial birds, such as quail deltacoronavirus (QdCoV) and munia coronavirus (MuCoV) (36, 37). Among various regions, Hong Kong, China, has exhibited the highest diversity of δ-CoVs identified in wild birds (38).

## DISTRIBUTION AND TRANSMISSION OF γ-CoVs AND δ-CoV AMONG BIRDS

With growing research into γ-CoVs and δ-CoVs, there is increasing emphasis on the interspecies transmission of these avian CoVs. The diversity of CoVs infecting wild birds and domestic waterfowl is remarkable. Avian taxa that commonly serve as reservoirs for these viruses include various waterbirds, such as *Anseriformes, Charadriiformes, Pelecaniformes, Suliformes*, and *Sphenisciformes*. Additionally, bird orders frequently interacting with humans, such as *Galliformes, Columbiformes*, and *Passeriformes*, have also been identified as carriers of these viruses. Furthermore, the presence of these viruses has been documented in *Gruiformes, Accipitriformes, Strigiformes, Falconiformes, Cathartiformes, Psittaciformes, Piciformes*, and *Otidiformes* (http://covdb.popgenetics.net/v3).

Drawing definitive conclusions from diverse studies (27, 39–44), representing different geographic locations, targeting various avian species, and employing distinct methodologies of varied quality to detect γ- and δ-CoVs can be challenging. Nevertheless, notable variations in the prevalence and genetic heterogeneity of γ- and δ-CoVs in terrestrial birds and domestic waterfowl have been observed. For instance, it has been shown that the RNA-dependent RNA polymerase (RdRp) gene sequences of γ- and δ-CoVs form distinct phylogenetic clades (44). Within this branch, the overall sequence identity of the RdRp gene differs by less than 7%. In contrast, δ-CoVs formed a distinct phylogenetic branch from γ-CoVs. Similarly, a recent study suggested that wild waterfowl are more susceptible to γ- and δ-CoVs, while δ-CoVs found in wild terrestrial birds exhibit close genetic relationships to known PDCoVs (45). This supports the hypothesis that wild waterfowl may serve as the natural reservoir host for δ-CoVs. However, further research is needed to determine whether the infection rates of CoVs in domestic and wild waterfowl are consistent. These findings align with previous investigations on CoV infection in avian species, including wild waterfowl and various other birds (37, 44, 46). Taken together, these findings suggest that γ- and δ-CoVs are widespread within the waterfowl population.

While Jordan et al. (47) suggest that wild waterfowl and shorebirds may not be significant natural hosts of CoVs in American birds, their prior research in Asia indicates that the detection rates of these viruses exhibit regional and temporal variability due to the species-specific nature of avian and aquatic fauna. It is important to note that CoVs can persist in wild birds and waterfowl for extended periods, causing subclinical infections similar to low-pathogenic avian influenza (42). However, the understanding of avian CoV distribution remains incomplete in many areas. The disparate infection rates observed among CoVs could potentially be attributed to the diverse habitats inhabited by birds and waterfowl across various countries and regions. Moreover, the global dissemination of γ- and δ-CoVs among wild avian populations and other animal cohorts may be facilitated by multiple migratory pathways, connecting avifauna in disparate countries and regions.

Notably, a serological study of wild birds in the Middle East found that 75% of tested falcons had specific antibodies against Falcon coronavirus (FalCoV UAE-HKU27) (48). This finding signifies the extensive prevalence of δ-CoVs within the falcon population, effectively eliminating the possibility of residual viral presence in fecal samples resulting from avian predation. It further suggests that raptors could play a pivotal role in the epidemiology of CoVs, highlighting the importance of the food chain as a possible route for transmission (49).

Mass migration of bird species alone cannot explain the widespread abundance of CoVs in waterfowl and wild bird populations. The transmission of CoVs in various waterfowl and wild birds may be achieved through a combination of multiple pathways. Recent research has revealed that the simultaneous infection of two distinct CoV genera (γ and δ) is prevalent among wild bird communities, as reported worldwide (Poland, Russia, and Australia) (27, 50, 51).

## GENETIC DIVERSITY AND RECOMBINATION EVENTS

Genetic recombination plays a pivotal role in the ecological adaptation of CoVs, possibly leading to shifts in tissue and host specificity (52). CoVs exhibit high rates of RNA recombination due to their RdRp, which can switch templates during RNA synthesis, resulting in the generation of novel viral strains (53). CoVs have been implicated in a range of diseases affecting the respiratory and digestive systems in both humans and animals. Among avian CoVs, the majority belong to the γ genus (54) and have been associated with various avian species, including chickens, turkeys, sparrows, ducks, geese, and pigeons. While previous studies have demonstrated recombination events between γ-CoVs in different bird species and β-CoVs in various mammals (48, 55), evidence for recombination between CoVs belonging to different genera remains limited.

Highly contagious viruses, like IBV, exhibit multiple serotypes, some of which arise from the accumulation of mutations in the S gene, similar to antigenic drift observed in influenza viruses (56). Additional serotypes are generated via homologous RNA recombination, enabling the genetic exchange of the S gene among distinct viral strains. Considering the crucial role of the S protein in mediating host cell attachment and its possession of virus-neutralizing epitopes, genetic recombination events involving the S gene have the potential to generate new strains or serotypes, potentially amplifying their pathogenicity in different host species. Given the widespread transmission of γ- and δ-CoVs in wild birds and domestic waterfowl, it is plausible that mutations and recombination events within their genomes have contributed to this phenomenon.

Analysis and identification of the dominant CdCoV/CK/USA/Beaudette/1937 and Ontario CdCoV/TK/MG10/2007 were performed prior to the comparative study of the dominant duck CoVs DdCoV/GD/2014 (23). The analysis revealed an abundance of substitutions and mutations within the 1ab gene of DdCoV/GD/2014, including 10 mutations that induced frameshifts. Similarly, Domańska-Blicharz et al. (36) observed the most significant variability within the segment encoding the N-terminal domain of the S protein, responsible for receptor binding, within the S gene of the Polish isolate QdCoV PL/G032/2015 and the Dubai isolate QdCoV UAE-HKU30. This segment exhibited substantial amino acid insertions at positions 96–99, 112–113, and 360–370, as well as solitary amino acid mutations at positions 233, 414, 1,171, 291, and 353. These genetic alterations disrupted the coding sequence of the viral genome, modifying the amino acid composition of the gene—an exceptional occurrence rarely observed in other viral species. However, as these mutations were not identified through consensus sequencing of the dominant virus population in current studies, it is important to acknowledge that some of these insertions may represent defective viruses lacking replication competence. Nevertheless, within a given population, stochastic mutations can arise due to the abundance of individuals, thereby presenting a vast reservoir of heritable diversity.

CoVs possess a distinctive ability for homologous RNA recombination, wherein viral RNA undergoes random template switching during replication. This intriguing phenomenon, termed “selective replication,” was first documented in a seminal study on murine CoVs in 1985 (57). The study revealed that concurrent infection with genetically similar viral strains resulted in an increased rate of recombination. Key factors contributing to this phenomenon include the vast genome of CoVs; their ability to segregate, recombine, and relocate RNA; and the presence of subgenomic lengthy chains that serve as templates for molecular switching (58). The replication phase of the virus may harbor the primary determinants for recombination. In their investigation, Wang et al. (59) discovered that CoVs produce subgenomic RNAs during replication, which play a crucial role in facilitating homologous recombination between genes originating from distinct lineages or other viruses.

Phylogenetic analysis of the replicase pp1ab gene sequences from the newly discovered DdCoV/GD/2014 revealed that DdCoV/GD/2014 and 11 CdCoV strains share a lineage based on the nsp5 and nsp16 gene sequences, suggesting that the nsp5 and nsp16 genes of DdCoV/GD/2014 may have originated from CdCoV. Further analysis using RDP software confirmed the occurrence of genomic recombination between the CdCoV and DdCoV lineages (23), highlighting the significant role of interspecies recombination in the evolution of novel CoVs, particularly between closely related viral lineages.

Phylogenetic analysis of four δ-CoVs isolated from wild birds in the United Arab Emirates revealed that QdCoV UAE-HKU30 had a close relationship with PDCoV HKU15 and sparrow deltacoronavirus (SpDCoV, HKU17) in the 3CLpro, RdRp, Hel, and N genes, while showing similarity to MuCoV HKU13 in the S gene (48). PDCoV HKU15 and Asian leopard cat coronavirus shared a close relationship with SpDCoV HKU17 in the 3CLpro, RdRp, Hel, and N genes but had a distant relationship in the S gene. They also exhibited a close relationship with the nightingale coronavirus (NHCoV_HKU19). Bootscan analysis further confirmed a recombination event in the S gene region of QuaCoV UAE-HKU30 (48). This suggests that the enhanced capacity for recombination among γ- and δ-CoVs contributes to their spread, persistence, and potential cross-bird species transmission.

## CROSS-SPECIES TRANSMISSION OF δ-CoV

In contrast to γ-CoVs, δ-CoVs show stronger associations with mammalian hosts. A 2021 study by Chu et al. (60) successfully identified δ-CoVs in fecal samples from black-headed gulls in Yunnan, China, suggesting that the host range of δ-CoVs includes at least 18 avian species and 2 mammalian species. Notably, PDCoV, frequently encountered in mammals, particularly pigs, appears closely related to avian δ-CoVs. These viruses have been found in both avian and mammalian populations, suggesting potential cross-species transmission.

In 2018, Wang et al. (59) conducted RT-PCR tests on bird feces collected from various lakes and wetlands in Hunan, China, and detected the presence of the δ-CoV RdRp gene. Preliminary analysis revealed significant similarity (40%) with other δ-CoVs previously identified in pigs and birds. Furthermore, in 2017, Chen et al. (61) discovered the SpDCoV strain in sparrow feces samples taken from a pig farm in the United States. Its sequence similarity to PDCoV and SpDCoV HKU17 surpassed that of other avian δ-CoVs, suggesting a plausible evolutionary connection between SpDCoV and PDCoV. Additionally, Woo et al. (33) found that SpDCoV HKU17 and PDCoV strains exhibit over 90% amino acid identity across the seven conserved domains of RdRp, indicating that they belong to the same species.

The emergence of PDCoV has been suggested to be linked to recombination events between different lineages of SpDCoVs (31). This hypothesis is supported by the close genetic similarity between PDCoV and certain avian δ-CoVs. Among avian CoVs, the δ-CoV found in sparrows and pigeons exhibit the highest genetic similarity to PDCoV, suggesting a potential role of these avian species in the evolution of PDCoV. Notably, human infections with PDCoV have also been reported, raising concerns about its potential zoonotic transmission (62).

The potential transfer of δ-CoVs from avian species to mammals, particularly pigs, remains a subject of ongoing debate. While some researchers propose an avian origin for δ-CoVs, suggesting subsequent transmission to mammals, Chen et al. (61) argued that the N sequences of PDCoV and SpDCoV share a degree of conservation in specific regions, potentially leading to cross-reactivity in PDCoV N gene-based PCR assays. This raises concerns about potential false-positive results in PCR testing for PDCoV in pigs. Consequently, comprehensive analysis of PDCoV RT-PCR-positive pig samples, particularly those potentially contaminated with avian droppings or other excreta, is essential. Serological data for PDCoV could provide evidence of infection in pigs. However, the high degree of conservation in the N protein between porcine and sparrow δ-CoVs necessitates the use of specific peptide fragments for definitive serological results to avoid cross-reactivity.

**Fig 1:**
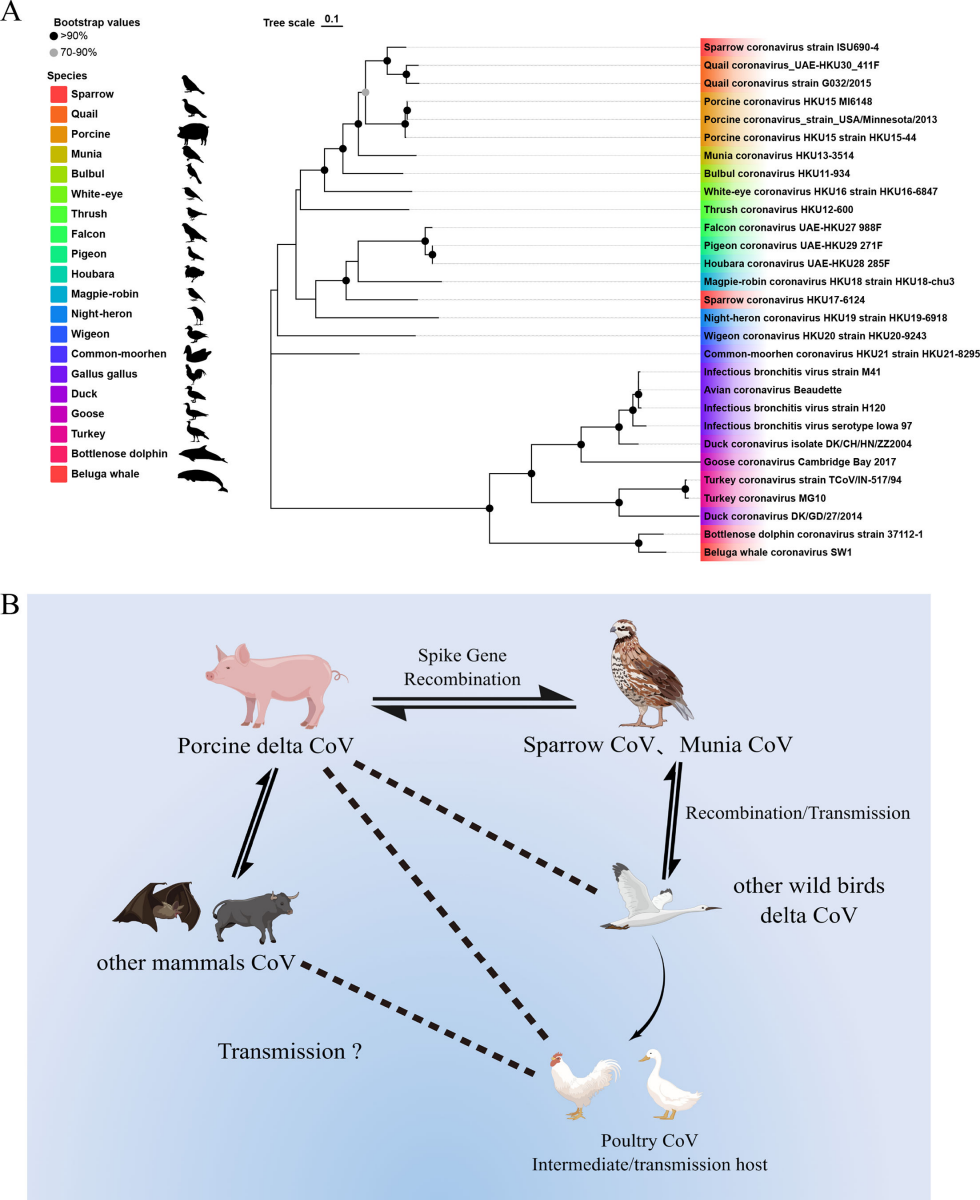
(**A**) This phylogenetic tree, constructed using the maximum likelihood algorithm (GTR+G+I model) in MEGA 7, depicts the evolutionary relationships between gamma- and delta-coronaviruses. The full genome sequences representing diverse representative strains from waterflow, wild birds, and mammals, deposited in GenBank, were included in the analysis. Branch colors indicate the species from which the sequences were sampled. The tree was visualized and processed using the Tree Visualization By One Table (tvBOT) version 2.6 online tool (https://www.chiplot.online/tvbot.html). (**B**) The possible interspecies transmission routes of δ-CoVs. The presence of PDCoV, SpDCoV, MuCoV, and various other mammalian or avian CoVs implies the inherent host range of CoVs with a double-headed arrow. In instances where complete genome sequences and/or experimental evidence is unavailable to substantiate the stated facts and direction, it is prudent to exercise caution and depict this uncertainty with a dotted line.

While the precise transmission routes remain unclear, the aforementioned findings support the likelihood of interspecies δ-CoV infection. Figure 1 illustrates the evolutionary relationships between representative γ- and δ-CoVs and graphically depicts potential transmission routes based on current reports. Continued surveillance and focused research are crucial to fully understand the ecology of δ-CoVs.

## ROLE OF RECEPTORS IN CROSS-SPECIES INFECTION OF γ-CoVs and δ-CoVs

The initial step in viral infection of host cells is the recognition and binding of cell receptors, a critical factor determining the virus’ host range. The S protein facilitates CoV attachment to both carbohydrate and protein receptors on cell surfaces. However, the receptors for CoVs in domestic waterfowl and wild birds remain largely unknown.

Previous research has revealed that IBV, similar to avian influenza A virus (AIV), binds to sialic acid in the host. The propagation of most IBV strains through introduction into the avian embryo’s allantoic cavity has been extensively documented (63). However, the reduced affinity of the S protein for sialic acid, coupled with its enhanced specificity, limits its ability to exhibit a broad host tropism, unlike AIV (64). Recent studies have investigated various cell receptors, such as aminopeptidase N (APN), shedding light on the cross-species properties of δ-CoVs (65).

For example, a 2018 study demonstrated that PDCoV can enter cells in both mammals and birds using APN as a receptor (66). Further supporting this notion, a 2021 study infected pigs with a chimeric virus constructed by combining the S protein or RBD of PDCoV and SpDCoV. This study observed a significant reduction in virulence and intestinal tropism, while viral replicability in the respiratory tract remained unchanged. Interestingly, when APN was knocked out in swine testis cells, the infectivity of the chimeric virus icPD-CoV-RBDISU increased, providing evidence supporting the hypothesis that δ-CoVs can utilize a wide range of receptors (67). These findings suggest that δ-CoVs’ ability to infect multiple bird and mammal species may be attributed to their capacity to employ a broad spectrum of receptors.

While δ-CoVs have been detected in certain mammals, including Asian leopard cats, Chinese ferret badgers, and other wild birds, their ability to replicate in these species remains unclear (28, 68). Importantly, virus isolation attempts for these CoVs in domestic waterfowl and wild birds, using avian embryos and various cell lines, have not been successful. The receptors for these viruses remain largely unknown.

## GENOME STRUCTURE AND SIMILARITY AMONG VARIOUS CoVs

**Fig 2:**
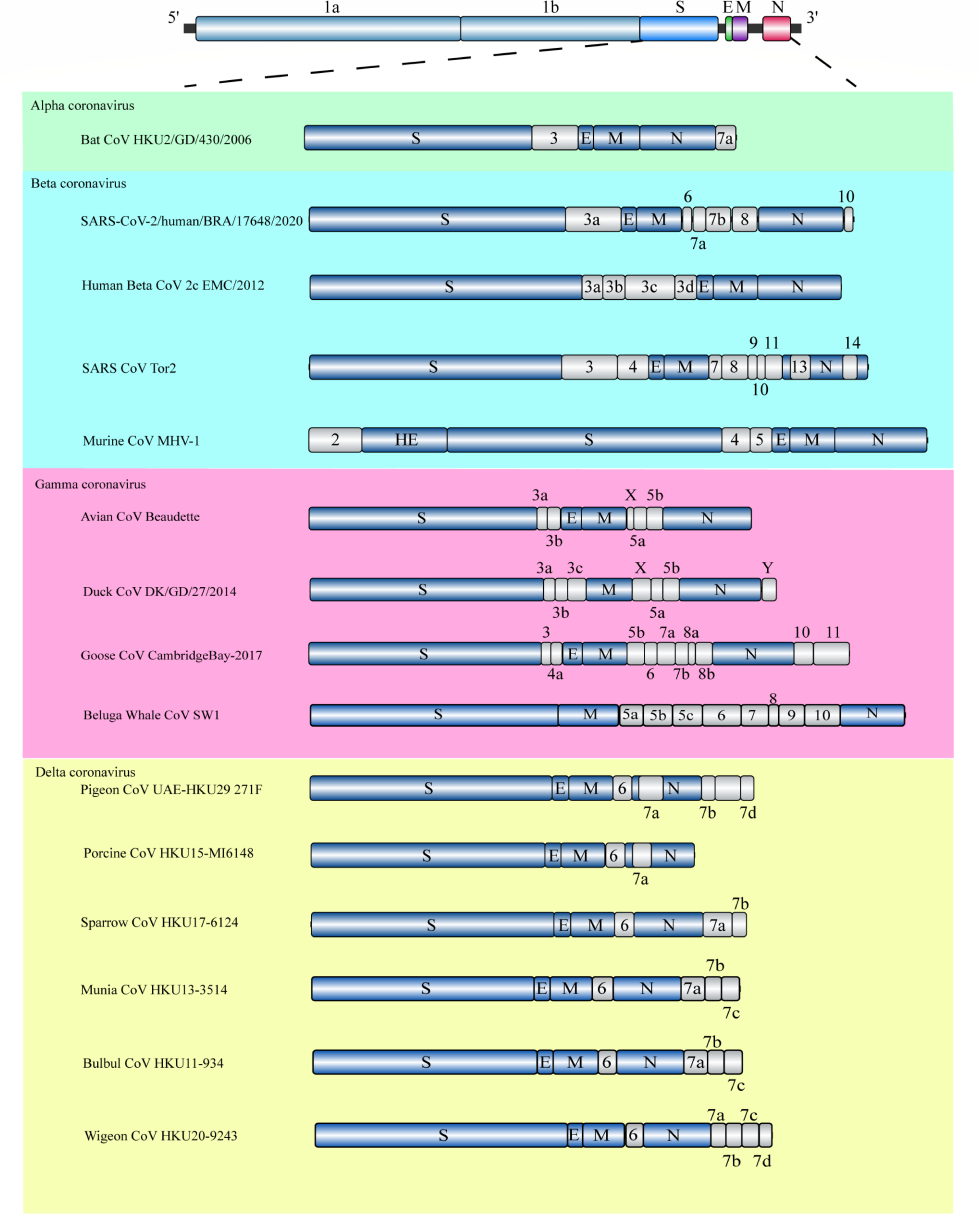
The chart contents reflect the comparison results of the genome structure of avian CoV Beaudette, duck CoVs DK/GD/27/2014, pigeon CoVs UAE-HKU29, porcine CoVs HKU15-M16148, sparrow CoVs HKU17-6124 and SARS-CoV-2/human/USA/V02071/2021, goose CoVs Cambridge_Bay_2017, human betacoronavirus 2c EMC/2012(MERS), SARS coronavirus Tor2, murine coronavirus MHV-1, and bat coronavirus HKU2/GD/430/2006. All genomic information presented was obtained from the NCBI GenBank database. The viral nucleotide sequence is annotated with colored arrow segments to indicate distinct open reading frames (ORFs). Notably, the primary ORFs of CoVs, namely, structural proteins and accessory proteins, are visually represented by blue and gray, respectively.

To comprehensively analyze gene information gathered from various avian and mammalian CoVs, a comparative gene structure diagram was generated using IBS 2.0 software (69) (Fig. 2). The genome sequences of these strains consistently consist of the open reading frames (ORFs) ORF1ab, S, E, M, and N, arranged in a 5′ to 3′ direction. ORF1ab, responsible for encoding non-structural proteins, occupies approximately 50% of the total genome size. While the genome sequences of the two γ-CoVs and three δ-CoVs mentioned above exhibit significant similarities, notable variations are observed in the latter half of the genome, particularly in the ns6 and ns7 genes responsible for encoding auxiliary proteins.

Previous studies have demonstrated that the ns7a protein of PDCoV can enhance membrane permeability in mammalian cells (70). These accessory proteins are believed to play crucial roles in viral infection, pathogenesis, and immune regulation within the host (71, 72). However, the specific contribution of these auxiliary proteins to the cross-species transmission of avian and wild bird CoVs remains unclear and warrants further investigation.

The genome of avian CoVs typically exhibits a conserved arrangement of ORFs: 1ab-S-3a-3b-E-M-4b-4c-5a-5b-N-6b (73). However, BcanCoVCB17 displays a unique genetic structure, featuring two additional ORFs (7a and 7b) located between the M and N ORFs and two further ORFs (10, 11) upstream of the N gene. Notably, these ORFs exhibit no significant similarity to known protein sequences found in conventional avian CoVs.

Comparative analysis of the amino acid sequences of the 1ab replicase polyprotein across seven conserved structural domains, comparing goose CoVs, avian CoVs, and beluga whale CoVs, revealed a similarity below 90%. This finding strongly supports the classification of goose CoVs as a distinct species, separate from avian CoVs.

Intriguingly, despite this distinct genetic architecture, goose CoVs share a commonality with typical avian CoVs: the presence of a transcriptional regulatory sequence at the terminus of their precursor sequence. This observation suggests a potential shared ancestral origin between goose CoVs and avian CoVs.

## FUTURE PERSPECTIVES

### Diagnostic methodologies

A significant limitation in comprehensively studying avian CoVs, including those affecting wild birds and domestic waterfowl, is the dearth of dedicated serological and molecular diagnostic methods. Current research largely relies on RT-PCR amplification of limited regions of the RdRp gene. Expanding the availability of novel diagnostic tools, such as pseudotyped virus neutralization assays specifically targeting these viruses, is crucial to thoroughly understand their ecology.

### Host–virus interactions

The precise structure and receptor binding mechanisms of the S protein RBD region in waterfowl CoVs remain elusive. Further investigation into these aspects, including the role of receptor-augmenting enzymes, is necessary. Gaining a deeper understanding of the receptors and complex molecular mechanisms governing the interaction between domestic waterfowl CoVs and their hosts, facilitated by the development of highly effective cell lines for virus isolation, is essential.

### Viral genome and replication

The complex interplay of non-structural and accessory proteins within the γ-CoVs and δ-CoVs in birds, as well as the recombination processes among diverse CoV genera, warrants further investigation. Elucidating the functional roles of these proteins and their contributions to viral replication, pathogenesis, and interspecies dissemination is crucial for developing effective control strategies.

### Genetic diversity and transmission

Augmenting bioinformatics resources and establishing a comprehensive CoV genome repository will facilitate a deeper understanding of the genetic diversity, variations, and interspecies transmission mechanisms of γ- and δ-CoVs in domestic waterfowl and wild birds. This will ultimately enhance our understanding of their cross-species transmission dynamics and potential implications for public health.

Overall, future research should prioritize the following:

- Development of novel diagnostic methods tailored for avian CoVs
- Comprehensive investigation of the S protein RBD region and its interactions with host receptors.
- Elucidation of the roles of non-structural and accessory proteins in viral replication and pathogenesis.
- Establishing a comprehensive genome repository and exploring the genetic diversity and interspecies transmission dynamics of avian CoVs.

By addressing these key areas, we can gain a more complete understanding of avian CoVs and their potential threats to human health and pave the way for more effective preventative and therapeutic strategies.

## Data Availability

The data sets used and/or analyzed during the current study are available from the corresponding author on reasonable request.
